# Nutritional Habits in 8–11-Year-Old Spanish Children According to Their Gender

**DOI:** 10.3390/nu17061016

**Published:** 2025-03-14

**Authors:** Josune Rodríguez-Negro, Silvia Sánchez-Díaz, Javier Yanci

**Affiliations:** 1 Research Group (GIKAFIT), Department of Physical Education and Sport, Faculty of Education and Sport, University of the Basque Country (UPV/EHU), 01007 Vitoria-Gasteiz, Spain; josune.rodriguez@ehu.eus; 2 Department of Education and Teacher Training, Faculty of Education, Legal Sciences and Humanities, European University of Madrid (UE), 28670 Madrid, Spain; silvia.sanchez@universidadeuropea.es; 3 AKTIBOki: Research Group in Physical Activity, Physical Exercise and Sport, Sports and Physical Exercise, 01007 Vitoria-Gasteiz, Spain

**Keywords:** Mediterranean diet, KidMed, nutrition, diet, healthy habits

## Abstract

**Objectives**: The main goals of this study were to describe the nutritional habits of 8–11-year-old children and to analyze the differences in nutritional habits according to their gender. **Methods**: A total of 240 children (114 boys and 126 girls) from a Spanish primary education state school completed the Mediterranean Diet Quality Index in Children and Adolescents (KIDMED) questionnaire. **Results**: The average score obtained on the KIDMED questionnaire for all participants was 7.16 ± 2.06 points, and only the 45.4% of the participants showed optimal adherence to the Mediterranean diet. No differences between boys and girls were found in their adherence to the Mediterranean diet (boys: 7.38 ± 1.95 vs. girls: 6.97 ± 2.14, *p* = 0.648). However, in the 10-year-old group, significant differences according to gender were found for some specific consumptions (i.e., olive oil, nuts, yogurts and cheese) and habits (i.e., skipping breakfast). **Conclusions**: The results obtained in this study seem to indicate that only half of the children have optimal adherence to the Mediterranean diet, so their dietary patterns should be strengthened. Moreover, some differences were found for some specific consumptions and habits in children older than 10 years old. These results could have important implications for families, schools and health promoters, since they have the potential to foster healthy nutritional habits. Furthermore, they underline the importance of gender-sensitive nutritional interventions in children older than 10 years old.

## 1. Introduction

The acquisition of healthy habits related to physical activity, sleep and nutrition during childhood is essential for children’s health [1,2]. Furthermore, healthy habits during childhood are crucial for maintaining these behaviors during adulthood and are linked to better lifelong health [3]. However, most children do not meet the international recommendations for healthy habits [4,5,6]. Healthy nutritional habits play a crucial role in children’s physical and mental health, as well as in their development and emotional well-being [7,8,9]. Despite the benefits of healthy habits during childhood, 93.75% of the studies identified a dietary pattern considered unhealthy [10]. Furthermore, unhealthy nutritional habits during childhood are related to a lower health-related quality of life [11], and they can set a trajectory of adverse health outcomes well into adulthood [12,13]. A healthy diet is a health-promoting and disease-preventing diet [14] that promotes balanced and nutrient-rich food [11], such as the Mediterranean diet.

The Mediterranean diet is characterized by a high intake of plant-based foods, such as fruits, vegetables, whole grains, nuts, seeds, legumes, etc., a moderate intake of ani-mal-based foods and olive oil as the principal fat source [15,16,17]. The Mediterranean diet is considered healthy, as children and adolescents who adhere to this diet have improved health outcomes [18]. Among the benefits of Mediterranean dietary patterns in children and adolescents, a lower risk of illness, improved psychosocial health and a better health-related quality of life [11,19,20,21] can be highlighted. Also, Mediterranean dietary patterns have been shown to be effective for maintaining a healthy weight and preventing obesity [22]. Furthermore, the systematic reviews of Grosso et al. [23] and Sánchez-Sánchez et al. [24] have concluded that the Mediterranean diet is inversely correlated with the risk of non-communicable chronic diseases, such as cardiovascular diseases and diabetes. Even though the Mediterranean diet has been the preferred dietary pattern traditionally in Spain [16], nutritional habits have significantly changed during recent years [25,26], and research has shown a decrease in Mediterranean diet adherence and healthy eating habits among children and adolescents [27,28]. Therefore, analyzing the variables that influence eating habits in children has aroused growing interest in the scientific literature.

A substantial body of literature has found differences in the dietary patterns of children, related to their own behavior and their parents’ educational and socioeconomic level, nutritional knowledge and parental sport participation [10,29,30]. A higher educational level of parental education showed a lower probability of high sugar consumption [31]; fathers having a higher educational level was related to greater Mediterranean diet adherence [30] and a lower educational level in mothers was related to higher snack consumption [32]. Some studies have also analyzed children’s dietary patterns according to gender [33,34,35], but the results are contradictory. On the one hand, Wall et al. [35], in a study carried out on 591 New Zealand children, observed that 7-year-old boys had healthier eating patterns (traditional diet) than girls. Similarly, Rodrigues et al. [34], in a study carried out on 1063 Portuguese children, also observed that 6–8-year-old boys had healthier diets (a higher consumption of the Portuguese diet) than girls. In contrast, Lee et al. [33] found that girls were healthier in a study carried out on 154 7–9-year-old Korean children. Oellingrath et al. [32], in a study carried out on 6–10-year-old children from Norway, also found that boys had higher consumption rates of snack and junk food consumption with high fat and sugar contents than girls. However, another study did not find differences in dietary patterns between boys and girls [36]. Therefore, there are no consensual results about the effects of gender on dietary habits, so it may be necessary to delve more deeply into this topic, especially in children.

Given the importance of nutritional habits during childhood, a myriad of research studies focus on whether school children follow dietary habits and recommendations. However, less is known about the differences in habits according to gender, as many articles have not divided their results according to gender [37], and in the few articles where gender analysis was carried out, the results are contradictory [32,33,34,35]. While this information might be important, the influence of gender on dietary habits during childhood has not been deeply explored yet, and evidence-based guidance is still needed. Therefore, the main goals of this study are to describe the nutritional habits of 8–11-year-old children and to analyze the differences in their nutritional habits according to their gender.

## 2. Materials and Methods

### 2.1. Participants

A total of 240 children (114 boys and 126 girls) between 8 and 11 years from a Spanish primary education state school were enrolled in this research. Table 1 shows the age, body height, body mass and body mass index (BMI) of all participants, divided by gender. Height and body mass were measured via a Stadiometer (SECA) in a standing position with shoes removed. The inclusion criteria were (1) not being sick the previous week and (2) being able to understand and answer the questionnaire. Before participation, participants and parents or legal guardians were informed about the aim and the design of the study, and the parents or legal guardians signed an informed consent form. The management team of the primary school to which the children belonged also approved the study. The study was performed in accordance with the Helsinki Declaration (2013) and was approved by the Ethics Committee (CEISH, code 2015/147) of the University of the Basque Country (UPV/EHU).

### 2.2. Procedure

In the present study, Mediterranean diet adherence of 8-to-11-year-old children was examined. To assess the Mediterranean diet adherence, the Mediterranean Diet Quality Index in Children and Adolescents (KIDMED) questionnaire [38] was administered to the participants in physical education sessions during school hours. The questionnaire was administered during the month of February, and a researcher, as well as the physical education teacher, were present on that day. Participants received an explanation about the questionnaire prior to administration, and all doubts were resolved. The questionnaire was answered in paper format, and the information was subsequently transferred to an Excel file.

### 2.3. Measures

Mediterranean Diet Quality Index in Children and Adolescents (KIDMED) questionnaire: The KIDMED questionnaire [38] was used to assess Mediterranean diet adherence among the participants. The KIDMED is a 16-item yes/no questionnaire. A total of 12 items had positive implications for the Mediterranean diet (+1 value), while four items had a negative meaning (−1 value). The final summed index ranged from 0 to 12 points and was classified into 3 grades according to the adherence to the Mediterranean diet (>8 optimal; 4–7 intermediate; <3 very low adherence). The KIDMED questionnaire has already been used in school-age children [39] and has been found to have good reliability values (Cronbach’s alpha = 0.79, 95% CI: 0.71–0.77, and κ = 0.66, 95% CI: 0.45–0.77) [40].

### 2.4. Statistical Analysis

The results are presented as means, standard deviations (SDs), frequencies and percentages (%). To determine the normality of the data and the equality of variances, the Kolmogorov–Smirnov statistic and the Levene test were used, respectively. The Student test for independent samples was used to determine whether any significant differences existed between genders in the questionnaire final score. The Chi^2^ statistical analysis was used to analyze the statistical significance in the distribution of frequencies and percentages of the answers for each gender. Data analysis was performed using the Statistical Package for Social Sciences (SPSS, version 23.0 for Windows, Chicago, IL, USA). Statistical significance was set at *p* < 0.05.

## 3. Results

**Figure 1:**
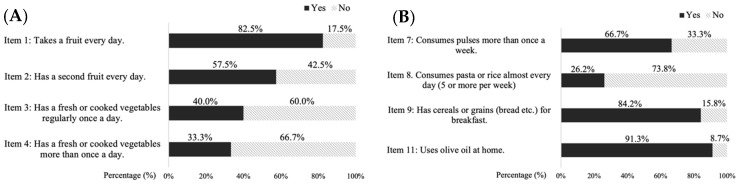
Consumption of fruits, vegetables (**A**), pulses, pasta, rice, cereals and olive oil (**B**).

The average score obtained in the KidMed questionnaire by all participants was 7.16 ± 2.06 points. On this KidMed, 45.4% of the participants showed optimal adherence to the Mediterranean diet (>8 points), 48.3% showed intermediate adherence (4–7 points), and 6.3% had low adherence (<3 points). If we analyze the results by item, the results for fruit and vegetable consumption (items 1–4) and the results of consumption of legumes, pasta, rice, cereals and olive oil (items 7–9, 11) are shown in Figure 1A and Figure 1B, respectively.

**Figure 2:**
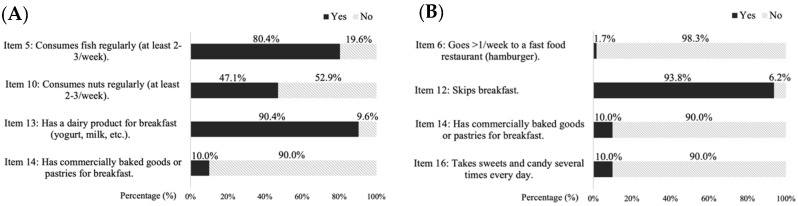
Fish, nuts and dairy product consumption (**A**) and unhealthy habits (**B**).

Figure 2 shows the results related to the consumption of fish, dairy products and nuts (2A: items 5, 10, 13, 14) as well as unhealthy habits such as going to fast food restaurants or eating sweets, candy and industrial pastries repeatedly (2B: items 6, 12, 14, 16).

Focusing on the gender of the participants, the 49.1% of the boys showed optimal adherence to the Mediterranean diet (>8 points), 47.4% showed intermediate adherence (4–7 points) and 3.5% showed low adherence (<3 points). In the case of the girls, 42.1% showed optimal adherence, 49.2% showed intermediate adherence and 8.7% showed low adherence. No differences between boys and girls were found in the adherence to the Mediterranean diet. The results obtained in each item of the KidMed questionnaire for boys and girls, as well as the differences between them, are presented in Table 2. No statistically significant differences between boys and girls were found in adherence to the Mediterranean diet (boys 7.38 ± 1.95 vs. girls 6.97 ± 2.14, *p* = 0.648).

**Figure 3:**
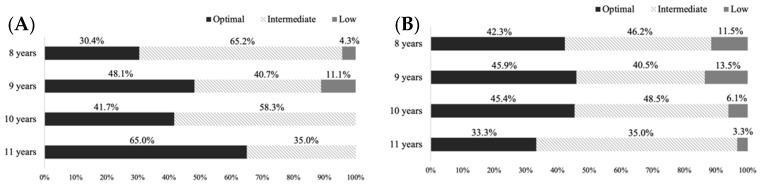
The adherence to Mediterranean diet for boys (**A**) and girls (**B**) at each age.

The results of the adherence to Mediterranean diet (i.e., optimal, intermediate or low) for each gender at each age are shown in Figure 3. No differences between boys and girls were found in the adherence to the Mediterranean diet at any age.

The results obtained in the KidMed questionnaire for boys and girls in each age group, as well as the differences between genders in each age group, are presented in Table 3. No significant differences between boys and girls in the KidMed total score was found in any age group: 8 years (boys 6.74 ± 2.13 vs. girls 6.73 ± 2.55, *p* = 0.636), 9 years (boys 7.22 ± 2.22 vs. girls 7.14 ± 2.28, *p* = 0.491), 10 years (boys 7.04 ± 1.82 vs. girls 6.85 ± 1.97, *p* = 0.707), nor 11 years group (boys 8.20 ± 1.47 vs. girls 7.11 ± 1.84, *p* = 0.415). Despire this, in the 10-year-old group, differences according to gender were found in item 11 (Uses olive oil at home), and in the 11-year-old group in item 10 (Consumes nuts regularly), item 12 (Skips breakfast) and item 15 (Takes two yogurts and/or 40 g of cheese daily).

## 4. Discussion

The primary objectives of this study were to describe the nutritional habits of children aged 8 to 11 years and to analyze the differences in nutritional habits according to their gender. A key contribution of this research is its focus on the differences in nutritional preferences between boys and girls. In addition, while many existing studies concentrate on the nutritional habits of adolescents or adults, there is a shortage of research specifically addressing the differences in nutritional habits among school-age boys and girls. Considering that lifelong eating habits are established during childhood [3], it may be relevant to know the nutritional habits in this population in order to be able to implement changes.

The KidMed questionnaire has been previously used to determine nutritional habits in different population groups, including children [39,40]. In the present study, the average score obtained in the KidMed questionnaire by all participants was 7.16 points. The KidMed score in the present study is higher than the scores obtained in other research, as some previous research with primary education children have a score between 4.7 and 5.9 points [41,42,43]. Furthermore, in the present study, 45.4% of the participants showed optimal adherence to the Mediterranean diet, 48.3% showed intermediate adherence and 6.3% showed low adherence. These results are significantly better than the ones obtained by Kanellopoulou et al. [43] in research performed with 1142 10–12-year-old children from Greece, where only 13.5% showed high adherence to the Mediterranean diet. Similarly, Grassi et al. [42] and Bonnaccorsi et al. [41] found that only 13.5% and 24.8% of the primary education students from Italy, respectively, had high adherence to the Mediterranean diet. Even though, our results are in line with Herrera-Ramos et al. [26], results in research performed with 1724 Spanish children between 8 and 12 years found that 45.5% of the children had high adherence to the Mediterranean diet, and only 8% had poor adherence. These differences in the adherence to the Mediterranean diet could be due to the country, as the present research, as well as the research of Herrera-Ramos [26], was conducted with Spanish children, while other research has been carried out in other European countries [41,42,43]. In fact, some previous research has stated that the Mediterranean diet has traditionally been the preferred dietary pattern in Spain [16,44]. Even if the results in Spain seem to be higher than in other countries, only one of two children have optimal adherence to Mediterranean diet, so dietary patterns should be strengthened through public health initiatives that promote children’s health [10,11].

In the present study, no significant differences between boys and girls were found in the adherence to the Mediterranean diet. Specifically, 49.1% of boys and 42.1% of girls showed optimal adherence to the Mediterranean diet, 47.4% of boys and 49.2% of girls showed intermediate adherence and 3.5% of boys and 8.7% of girls showed low adherence. Even if a large body of literature has been devoted to investigating the nutritional habits in children, no consensual results have been found when comparing genders. Some studies have concluded that girls have better diet quality and adherence to the Mediterranean diet [32,33,44], while others have stated that boys have better adherence [34,35]. Moreover, in line with our results, some other studies did not find differences in dietary patterns and adherence to the Mediterranean diet between boys and girls [42,45]. For example, Basiak-Rasała et al. [46] found some differences among genders in research carried out with 6–17-year-old Polish children and adolescents. Specifically, boys consumed carbonated beverages and fast-food meals more often than girls, and girls consumed fruit and drank mineral water more often than boys. However, these authors did not present the results for each gender divided by age. Similarly, Herrera-Ramos et al. [26] investigated the dietary habits in 8–16-year-old Spanish children and adolescents, but they did not present the gender differences by age, making it impossible to know if gender differences appear in all ages or just in some. There is a shortage of manuscripts that analyze the differences in nutritional habits according to gender at each age. Therefore, it could be interesting to know the gender differences at each age.

Even if in the present study no differences in the adherence to the Mediterranean diet were found between boys and girls, some differences have been found in specific consumptions at some ages. If we focus on the gender differences at each age, the results of this study showed that young boys and girls (8–9 years) have the same dietary patterns, but that older children (>10 years) have gender differences in some specific consumptions (i.e., olive oil, nuts, yogurts and cheese) and habits (i.e., skip breakfast). For example, 10-year-old girls use more olive oil at home than boys, and 11-year-old boys consume more nuts, yogurts and cheese than girls. They also skip breakfast more than girls. These results are in line with previous research that did not find differences in nutritional habits in young children (<9 years) [42,45,47], but found differences with older children (>10 years) [44,48]. For example, Grassi et al. [42] did not find differences in Mediterranean diet adherence between boys and girls of 6–8 years old from Italy, but Grams et al. [44] found that 10–13-year-old girls have better diet quality than boys. This seems to indicate that while there are no significant differences in food intake and habits between boys and girls in early childhood, some differences start to emerge around 10 years of age. These gender differences in dietary habits that emerge in preadolescents could be due to external and internal motivators, such as gains in physical performance, being thin to fit traditional norms or gaining autonomy [49]. Therefore, they underline the importance of gender-sensitive nutritional interventions that consider the gender gap when they are addressed to children older than 10 years old [50,51].

Despite the contribution this study can offer to understanding the nutritional habits of primary education boys and girls, it has certain limitations that need to be acknowledged. The research relied on a convenience sample, with all participants drawn from a single primary school, limiting the generalizability of the findings to a broader population. Furthermore, the results are based on the consumption declarations made through a questionnaire. However, it also has notable strengths, including the use of validated questionnaires specifically designed for child populations. These limitations underscore the need for further research on the topic and to elucidate the reasons for eating habits during childhood.

## 5. Conclusions

In conclusion, the results obtained in this study appear to indicate that only half of the children demonstrate optimal adherence to the Mediterranean diet. Although the results of this and some other Spanish samples appear to be higher than those obtained in other countries, dietary patterns should be strengthened. This study revealed no gender disparity in adherence to the Mediterranean diet among children over 10 years of age. However, it did identify specific variations in dietary intake, including higher consumption of olive oil, nuts, yogurt and cheese and skipping breakfast among children in this age group. These findings underscore the significance of promoting healthy nutritional habits within families, educational institutions and health promotion initiatives. Furthermore, the results emphasize the necessity for gender-sensitive nutritional interventions for children older than 10.

## Figures and Tables

**Table 1:** Participants’ characteristics.

	Age (year)	Body Mass (kg)	Height (cm)	BMI z-Score
All	9.65 ± 1.16	36.54 ± 9.06	139.50 ± 8.46	0.9
Boys	9.75 ± 1.21	35.33 ± 7.39	139.24 ± 7.83	0.7
Girls	9.56 ± 1.11	37.60 ± 10.23	139.73 ± 9.01	1.1

BMI = body mass index.

**Table 2:** Description of nutritional habits with the KidMed questionnaire for boys and girls.

	Boys (*n* = 114)	Girls (*n* = 126)	Chi^2^ (*p* Value)
	Yes *n* (%)	Yes *n* (%)	
Item 1: Consumes a fruit every day.	91 (79.8%)	107 (84.9%)	1.077 (0.299)
Item 2: Has a second fruit every day.	71 (62.3%)	67 (53.2%)	2.031 (0.154)
Item 3: Has a fresh or cooked vegetables regularly once a day.	48 (42.1%)	48 (38.1%)	0.401 (0.527)
Item 4: Has a fresh or cooked vegetables more than once a day.	44 (38.6%)	36 (28.6%)	2.707 (0.100)
Item 5: Consumes fish regularly (at least 2–3/week).	94 (82.5%)	99 (78.6%)	0.574 (0.449)
Item 6: Goes >1/week to a fast-food restaurant (hamburger).	3 (2.6%)	1 (0.8%)	1.234 (0.267)
Item 7: Consumes pulses more than once a week.	74 (64.9%)	86 (68.3%)	0.301 (0.583)
Item 8: Consumes pasta or rice almost every day (5 or more/week)	32 (28.1%)	31 (24.6%)	0.372 (0.542)
Item 9: Has cereals or grains (bread, etc.) for breakfast.	97 (85.1%)	105 (83.3%)	0.138 (0.710)
Item 10: Consumes nuts regularly (at least 2–3/week).	60 (53.6%)	53 (42.1%)	2.683 (0.101)
Item 11: Uses olive oil at home.	102 (89.5%)	117 (92.9%)	0.858 (0.354)
Item 12: Skips breakfast.	110 (96.5%)	115 (91.3%)	2.785 (0.095)
Item 13: Has a dairy product for breakfast (yogurt, milk, etc.)	104 (91.2%)	113 (89.7%)	0.165 (0.685)
Item 14: Has commercially baked goods or pastries for breakfast.	10 (8.8%)	14 (11.1%)	0.364 (0.546)
Item 15: Consumes two yogurts and/or some cheese (40 g) daily.	53 (46.5%)	50 (39.7%)	1.133 (0.287)
Item 16: Consumes sweets and candy several times every day.	13 (11.4%)	11 (8.7%)	0..475 (0.491)

**Table 3:** Description of nutritional habits using the KidMed questionnaire for boys and girls in each age group.

		8 Years		9 Years		10 Years		11 Years	
		Yes	Chi^2^ (*p* Value)	Yes	Chi^2^ (*p* Value)	Yes	Chi^2^ (*p* Value)	Yes	Chi^2^ (*p* Value)
Item 1: Consumes a fruit every day.	Boys	15 (65.2%)	1.154 (0.218)	22 (81.5%)	0.297 (0.586)	21 (87.5%)	0.729 (0.393)	33 (82.5%)	1.796 (0.180)
	Girls	21 (80.8%)		32 (86.5%)		26 (78.8%)		28 (93.3%)	
Item 2: Has a second fruit every day.	Boys	13 (56.5%)	0.525 (0.469)	18 (66.7%)	0.644 (0.422)	12 (50.0%)	0.013 (0.910)	28 (70.0%)	1.327 (0.249)
	Girls	12 (46.2%)		21 (56.8%)		17 (51.5%)		17 (56.7%)	
Item 3: Has a fresh or cooked vegetables regularly once a day.	Boys	12 (52.2%)	0.023 (0.879)	8 (29.6%)	0.052 (0.819)	7 (29.2%)	0.324 (0.569)	21 (52.5%)	0.577 (0.448)
	Girls	13 (50.0%)		10 (27.0%)		12 (36.4%)		13 (43.3%)	
Item 4: Has a fresh or cooked vegetables more than once a day.	Boys	8 (34.8%)	0.000 (0.990)	9 (33.3%)	0.627 (0.429)	10 (41.7%)	1.295 (0.255)	17 (42.5%)	1.147 (0.284)
	Girls	9 (34.6%)		9 (24.3%)		9 (27.3%)		9 (30.0%)	
Item 5: Consumes fish regularly (at least 2–3/week).	Boys	16 (69.6%)	0.097 (0.755)	22 (81.5%)	0.058 (0.809)	22 (91.7%)	1.733 (0.188)	34 (85.0%)	0.036 (0.850)
	Girls	17 (65.4%)		31 (83.8%)		26 (78.8%)		25 (83.3%)	
Item 6: Goes > 1/week to a fast-food restaurant (hamburger).	Boys	1 (4.3%)	1.154 (0.283)	2 (7.4%)	2.829 (0.093)	0 (0.0%)	0.740 (0.390)	0 (0.0%)	NV (NV)
	Girls	0 (0.0%)		0 (0.0%)		1 (3.0%)		0 (0.0%)	
Item 7: Consumes pulses more than once a week.	Boys	10 (43.5%)	3.305 (0.069)	17 (63.0%)	0.378 (0.539)	14 (58.3%)	0.415 (0.520)	33 (82.5%)	2.337 (0.126)
	Girls	18 (69.2%)		26 (70.0%)		22 (66.7%)		20 (66.7%)	
Item 8: Consumes pasta or rice almost every day (5 or more/week).	Boys	10 (43.5%)	2.310 (0.129)	7 (25.9%)	0.317 (0.574)	6 (25.0%)	0.865 (0.352)	9 (22.5%)	0.162 (0.687)
	Girls	6 (23.1%)		12 (32.4%)		5 (15.2%)		8 (26.7%)	
Item 9: Has cereals or grains (bread, etc.) for breakfast.	Boys	17 (73.9%)	0.060 (0.807)	23 (85.2%)	0.872 (0.350)	23 (95.8%)	1.099 (0.295)	34 (85.0%)	1.176 (0.278)
	Girls	20 (76.9%)		28 (75.7%)		29 (87.9%)		28 (93.3%)	
Item 10: Consumes nuts regularly (at least 2–3/week).	Boys	11 (47.8%)	0.023 (0.879)	19 (70.4%)	1.746 (0.186)	6 (25.0%)	0.830 (0.362)	24 (60.0%)	7.675 (0.006) **
	Girls	13 (50.0%)		20 (54.1%)		12 (36.4%)		8 (26.7%)	
Item 11: Uses olive oil at home.	Boys	19 (82.6%)	0.028 (0.868)	24 (88.9%)	0.001 (0.970)	20 (83.3%)	5.915 (0.015) *	39 (97.5%)	0.761 (0.383)
	Girls	21 (80.8%)		33 (89.2%)		33 (100%)		30 (100%)	
Item 12: Skips breakfast.	Boys	22 (95.7%)	0.008 (0.929)	26 (96.3%)	0.517 (0.472)	23 (95.8%)	0.100 (0.752)	39 (97.5%)	4.390 (0.036) *
	Girls	25 (96.2%)		34 (91.9%)		31 (93.9%)		25 (83.3%)	
Item 13: Has a dairy product for breakfast (yogurt, milk, etc.)	Boys	19 (82.6%)	0.341 (0.559)	25 (92.6%)	0.213 (0.645)	22 (91.7%)	0.212 (0.645)	38 (94.9%)	0.088 (0.766)
	Girls	23 (88.5%)		33 (89.2%)		29 (87.9%)		28 (93.3%)	
Item 14: Has commercially baked goods or pastries for breakfast.	Boys	2 (8.7%)	0.508 (0.476)	5 (18.5%)	0.767 (0.381)	2 (8.3%)	0.212 (0.645)	1 (2.5%)	0.726 (0.394)
	Girls	4 (15.4%)		4 (10.8%)		4 (12.1%)		2 (6.7%)	
Item 15: Consumes two yogurts and/or some cheese (40 g) daily.	Boys	9 (39.1%)	0.107 (0.744)	16 (59.3%)	0.706 (0.401)	7 (29.2%)	2.154 (0.142)	21 (52.5%)	6.076 (0.014) *
	Girls	9 (34.6%)		18 (48.6%)		16 (48.5%)		7 (23.3%)	
Item 16: Consumes sweets and candy several times every day.	Boys	2 (8.7%)	0.016 (0.898)	6 (22.2%)	1.542 (0.214)	0 (0.0%)	1.507 (0.220)	5 (12.5%)	0.106 (0.745)
	Girls	2 (7.7%)		4 (10.8%)		2 (6.1%)		3 (10.0%)	

* *p* < 0.05, ** *p* < 0.01 = significant differences between boys and girls; NV = no value.

## Data Availability

The original contributions presented in the study are included in the article, further inquiries can be directed to the corresponding author.
